# Music Therapy in the Treatment of Dementia: A Review Article

**DOI:** 10.7759/cureus.36954

**Published:** 2023-03-31

**Authors:** Apurv Shirsat, Roshan K Jha, Priyanshu Verma

**Affiliations:** 1 Anatomy, Jawaharlal Nehru Medical College, Datta Meghe Institute of Higher Education and Research, Wardha, IND; 2 Biochemistry, Jawaharlal Nehru Medical College, Datta Meghe Institute of Higher Education and Research, Wardha, IND

**Keywords:** quality of life, depressive state, dementia, music therapy, meta-analysis

## Abstract

The etiology of depression is the degeneration of the brain cells involved in cognitive function before the other brain cells. It is characterized by a neurological condition that causes a reduction in terms of physical, social, and cognitive impairment and has no cure presently. These nonpharmacological approaches, such as music therapy, enhance living outcomes for those dealing with dementia and also reduce behavioral incidence. Among these strategies is music therapy, and individual or gap-time psychological and educational counseling. Many scientists believe in the advantages of music for the brain. The brain is affected by music function and enhances some cognitive abilities, including the mechanism of speech, alteration, memory, and learning. Music can activate the limbic system, subcortical circuits, and emotionally related systems, inducing the sensation of well-being. The music itself is quite effective at increasing cerebral plasticity. Music therapy has powerful stimulation for neuroplastic alterations in the adult and developing brain. Dementia can be cured by music therapy and music-based intervention (nonpharmacological intervention) rather than by medication. This study highlights dementia therapy utilizing the music therapy method.

## Introduction and background

The usage of customized music playlists in medical settings to address mental and behavioral issues and symptoms of patients affected by dementia is on the rise. However, little is known about how individuals with various histories of mental illness and symptoms respond to music in various ways [1]. There has been an uptick in recent years sharp rise in the interest of the public in the healing results of music for patients affected by dementia. A music therapist's duties typically involve helping and instructing staff members, families, volunteers, and even certain teachers in the use of music-therapeutic approaches in addition to dealing with clients on a clinical level. Six researchers in music therapy (MT) from six different nations concurred that the time was right to host a roundtable where they could exchange their expertise in dementia care, or skill sharing in dementia care, and their indirect MT practice [2-7]. Music is frequently included in everyday routine as an adjuvant therapy to drug treatment, per national dementia plans in many different nations. However, facilities for long-term care capacity to characterize music-based interventions and treatment therapies is not given enough consideration [3]. An important global public health concern is dementia care. One of the toughest tasks in this situation is managing behavioral psychological symptoms of dementia (BPSD). Nonpharmacological approaches like music-based interventions, which are regarded as low-risk, accessible, and inclusive, appear like promising choices. This scoping research intended to map every music-based intervention utilized in dementia care, with a focus on BPSD, and debrief its elements, framework, and logic. Activities involving therapeutic music, such as MT, were included [4]. Degeneration in cognitive, behavioral, and emotional functioning characterizes rearrangement interventions for dementia as a clinical condition with several underlying causes. Pharmaceutical treatments are accessible to treat some of the symptoms of the syndrome, notwithstanding their limited efficacy. Very few studies were reported in the past on nonpharmacological remedies [5]. People with severe dementia have proven that multisensory stimulation and custom music are effective at managing their psychological and behavioral symptoms.

Due to the physical, psychological, financial, and social effects that dementia has on the elderly, their families, and their carers, it is regarded as a public health concern. Healthcare professionals might use MT as an additional treatment to address this problem [6]. Dementia, a major cognitive disability, is characterized by reminiscence loss. It has an impact on a person's behavior and emotions, which can harm their well-being and quality of life. According to studies, there's been an upsurge in motivation using music as a newer kind of treatment for dementia during the past few decades [7]. The term dementia overall includes conditions marked by advancement rearrangement that affects cognitive processes like remembering and language, in addition to behavioral changes like anxiety and sadness. To be able to treat dementia, both pharmacological and nonpharmacological interventions are used by consultants worldwide. Pharmacological interventions such as acetylcholinesterase inhibitors are used in such cases. Although there is medication for dementia, its potential benefits are minimal, especially in noncognitive outcomes. There is an increasing prevalence of dementia. MT, for example, is a nondrug approach that might produce superior outcomes [8]. The most common cause of disability in aged persons on a global scale is dementia. Treatment of patients affected by dementia may be difficult for clinicians due to the illness's various psychological and behavioral signs (BPSD). To control BPSD and prevent side effects linked to antipsychotic medication, the dementia action network along with the Beers Criteria of the American Geriatrics Society advocates nonpharmacological and behavioral interventions as a first-line treatment [9].

## Review

Methodology

**Figure 1:**
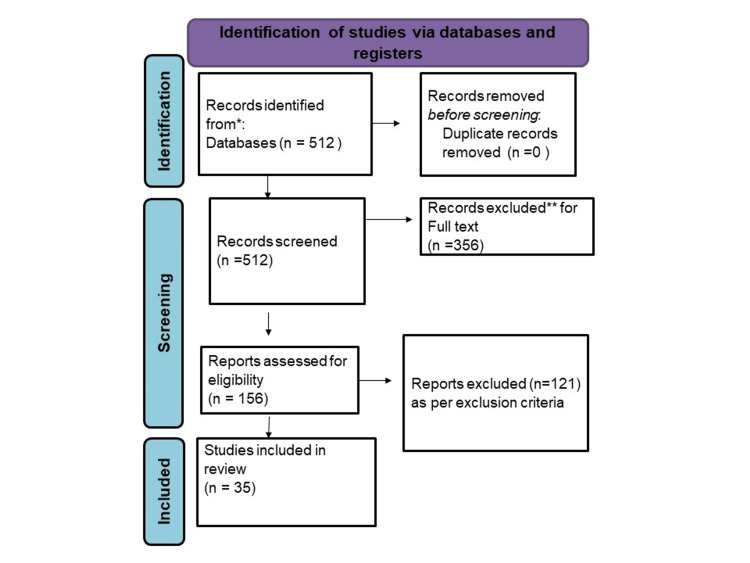
PRISMA flow diagram. Figure credits: Apurv Shirsat. PRISMA, Preferred Reporting Items for Systematic Reviews and Meta-Analyses

We undertook a systematic search through PubMed and CENTRAL in November 2020 using keywords such as "Music Therapy" and "Dementia" ([Title/Abstract]) OR ((Music Therapy(Title/Abstract))) OR (MT*[Title/Abstract]) OR ("Music Therapy" [MeSH Terms]) AND (" Dementia" [Title/Abstract]) OR (Dementia [Title/Abstract]) OR ("Dementia" [MeSH Terms]). We additionally searched for key references from bibliographies of the relevant studies. The search was updated in February 2022. One reviewer independently monitored the retrieved studies against the inclusion criteria, in the beginning, based on the title and abstract and then on full texts. Another reviewer also reviewed approximately 20% of these studies to validate the inclusion of studies (Figure *1*).

 Effects of dementia on the elderly population

**Figure 2:**
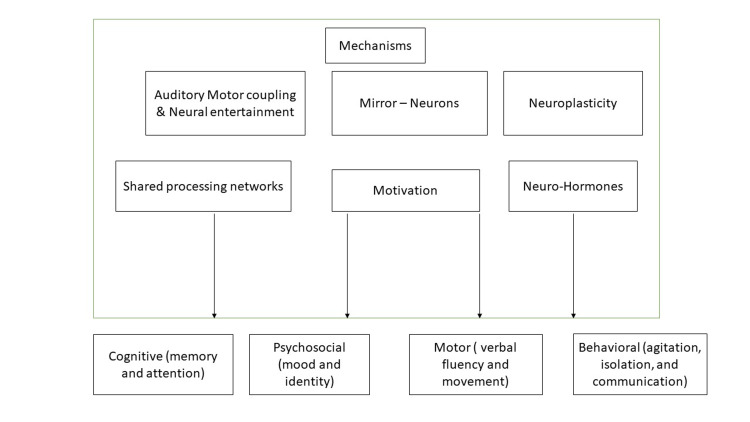
Mechanisms of music therapy in the treatment of dementia. Figure credits: Apurv Shirsat.

A neurological disorder called dementia is defined as a decline in emotional, social, behavioral, and cognitive abilities. Although pharmaceutical treatments are available, many of their effects on symptoms are brief to the disease. Numerous research has suggested using MT along with pharmaceutical treatment to lessen the effects of aging-related cognitive decline and behavioral disorders [10-13]. Agitation is a general term that denotes a variety of actions, such as behavior, agitation, wandering, and aggressive actions, which are the signs of common concern in people affected by dementia. Agitation decreases the likelihood of fruitful social interaction, which enhances organizational and mental exhaustion. Despite the prevalence of medical therapies, complementary or alternative approaches are still necessary. A possible approach to lessen agitation in people affected by dementia is music intervention [11]. Both domestically and globally, MT is frequently utilized informally in a residential setting facility to strengthen the communication and emotional, cognitive, and behavioral abilities of older patients having dementia [12]. Dementia is a catch-all word for numerous chronic conditions, including Alzheimer's, which has an impact on problem-solving, language, memory, and thinking ability, and interferes with daily activities. People affected by dementia frequently struggle with social and communication skills, which significantly affects both their quality of life and that of others around them (Figure 2).

In the past, Indians, Arabs, and Greeks knew about MT. In India, the literature on this technique is found in the Gandharva, Tattvas, and Raga Chikitsa (meaning raga treatment). Similar to how western music affects emotions, Indian classical culture is also available in Sangit Sudha. The first record of MT dates to a 1789 article in a Colombian journal titled *Music Physically Considered*. It was conducted in the 1800s. Wilhelm van de wall was the first to utilize MT in state-funded facilities (in 1936). At the University of Alberta, researchers took 42 children on trial aged 3 to 11 years and discovered that in individuals who listened to soothing music, the pain was reduced and distress was less compared with the patients who did not listen to music. People affected by dementia respond best to music when individualized music or melodies have resonance with them and are meaningful to them personally.

The study reported that musicians were 64% less likely to develop mild cognitive impairment (MCI) or dementia. The impact of these investigations on cognitive and physiological studies is minimal, despite the fact that some limitations of the results are consistent with MT's effectiveness in treating behavioral and psychological symptoms of dementia (BPSD), which are common. Dementia is already a critical issue worldwide where the human race is super-aging. This circumstance necessitates the development of rehabilitative strategies for relieving the symptoms of the patients. The goal of this study was to conduct a meta-analysis of the effects of MT on cognitive capacities in people affected by dementia [13]. According to reports, personalized music improves attitude and mood in people affected by dementia [14].

This study compared the behavioral expressions of passing happiness and pleasant behavior toward important people in MT and normal social situations [15]. Several studies were conducted to determine whether MT in people with dementia affects cognitive function, life quality, and depression. The impact of performing music therapies was assessed in randomized controlled trials on cognitive performance, psychological health, and social engagement in older persons with likely MCI or dementia [16]. A few studies also aimed to assess whether MT is beneficial in lowering distress in people with dementia [17].

**Figure 3:**
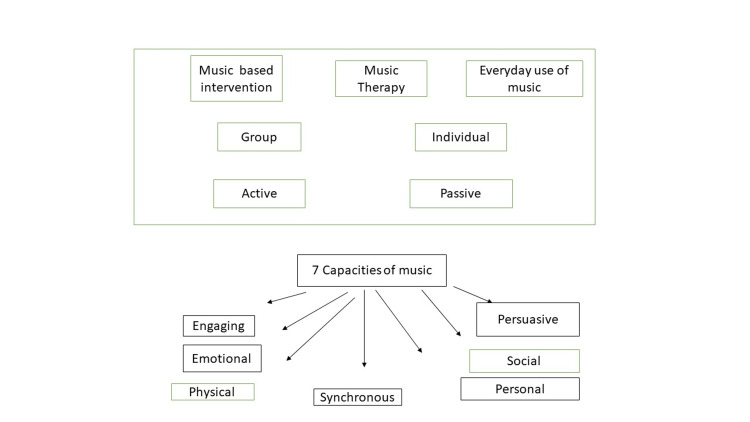
Effects of music therapy on behavior. Figure credits: Apurv Shirsat.

People with dementia are increasingly using music as a therapeutic aid. Numerous aspects of music are responsible for its positive impacts. We developed the Music, Memory, and Movement (MMM) course and assessed its effectiveness based on the recognition of seven therapeutic qualities of music. Utilizing music as a form of therapy for people with dementia has three key benefits. First, making use of music in treatment is convenient. Music is more accessible today than ever before, especially with recent technological advancements. In a variety of circumstances, from private music listening on iPods to public music listening, individuals have access to millions of songs spanning cultures and time. As people can participate in the experience (either through listening, moving, or generating music) regardless of their degree of performance, music is suitable for the dementia population (Figure *3*).

Discussion

People with dementia are as diverse as those who do not have dementia, and they each have their own life experiences, preferences, and histories. Each person's identity, history, and significant moments from their past are discovered to be intimately correlated with their musical tastes [18]. The standard of interaction between both the dementia patient and their family caregiver can be severely compromised by dementia progression, which can worsen feelings of loneliness and isolation. The findings of this comprehensive analysis show how music can enhance a caring relationship [19]. The belief that music is merely for enjoyment rather than being a useful and important instrument in the treatment of dementia was one of the biggest obstacles to the program's implementation. However, the straightforward form used to record mood and responsiveness to progressive multifocal leukoencephalopathy (PML) gave each MT a chance to consider the efficacy of the intervention, which encouraged widespread use [20].

Two simultaneous cluster randomized controlled trials are being carried out in this study using an adaptive study design. Three main aspects of the adaptive design are tested: different implementation methods, boosting enrollment of residents for whom the intervention will probably be beneficial, and smoothly running a stage three/four trial [21]. Although more work is required to take into account the disease's clinical complexity and build stronger evidence capable of addressing rehabilitative techniques, the protocol for the use of music played by the individual to bring back memories of past may represent an initial application of personalized medicine in dementia [22]. Unfortunately, a few characterizations are utilizing musical interventions, especially regarding the training of the practitioner and specifics regarding the use of music, in different studies and systematic opinions on the impacts of music like MT [23].

Although there is no direct evidence that MT has efficacy on the cognitive and behavioral condition of these patients, the results of some studies assert that listening to music has a beneficial impact on people with dementia [24,25]. Systematic reviews show that the only nonpharmacological interventions shown to greatly reduce behavioral disturbances (including depressive symptoms) in people affected by dementia are MT and behavioral therapy techniques, such as caregiver reinforcing desired behavior combined with appropriate training [24]. In this study, communication within the music-for-life group intervention for people with dementia in residential care was conceptualized. In doing so, it made an effort to comprehend how people with dementia communicate throughout an eight-week music-based activity [25].

Understanding how music-based therapies affect cognitive function favorably requires taking into account the different sorts of interventions. The concept of receptive and active music-based interventions could be used to categorize all music-based interventions. Participants in receptive interventions were required to listen to music, while those in active interventions were required to play percussion music, play instruments, or move to the music [26]. According to the literature, patients affected by Alzheimer's dementia may be able to avoid or delay the development of decreased quality of life by using unorthodox, nonpharmacological therapies as alternative therapies [27]. The main objective of this study is whether MT affects cognitive ability, general well-being, and melancholy state of mind. Compared to the therapies assessed in earlier studies, our study additionally includes a higher dose of tailored music-listening sessions, continual playlist review, and systematic selection of self-relevant music with each participant. By carefully observing the implementation process and assessing the implementation's performance, we also overcome the shortcomings of earlier studies [28].

The results showed that compared to the MT listening group or television control group, the brief group singing for MT directed by a music therapist had a greater impact on the quality of life and people with dementia [29]. This study's objective was to determine if the carers of people affected by dementia would accept and consider a personalized music-based intervention useful when it was introduced by a community-based organization [30]. The study's findings provide the first proof that the Modigliani-Miller theorem personalized music program may be linked to lowered levels of antipsychotic and sedative drug use as well as a decrease in BPSD among null hypothesis (NH) citizens with Alzheimer's disease and related dementia (ADRD) [31].

In line with previous research, older adults who engaged in interactive music assistance that included personalized music and activities, like clapping and dancing, experienced a greater reduction in the psychological and behavioral signs of dementia than older adults in a control group who received no music [32,33]. We examined how a musical dual-task training (MDTT) program affected those who have moderate-to-mild dementia. We created the MDTT with the expectation that focusing participants' attention during dual-task situations will enhance their cognitive and physical abilities. Our main findings showed that compared to the control intervention, eight separate MDTT sessions, each lasting 60 minutes, significantly improved attention regulation [34]. The majority of the research utilized tests to evaluate particular cognitive skills like memory and attention; executive function, language, and visuomotor skills; as well as general cognitive screening evaluations. Additionally, multifunctional batteries with measures for assessing behavior, mood, and particular protocols for MT evaluation were used [35].

We have detailed an innovative program that includes both caregivers and those who have younger-onset dementia (YOD). It combines online delivery of psychological approaches and evidence-based therapeutic songwriting strategies with trained facilitators to enhance social ties and mental health. We anticipate that if the program is a success, it will be simple to scale up and be able to help additional YOD dyads [36]. Dementia is already a serious problem worldwide, and society ages extremely quickly. The development of rehabilitation strategies is necessary for this situation of relieving the symptoms of patients. Psychiatric outcomes and cognitive functioning were the questions asked most frequently in the research, and global quality of life was based on general outcomes.

## Conclusions

Music could be a powerful treatment strategy. It is much needed to develop a clinical trial aimed to design standards based on the severity of dementia and methods that are compatible with existing pharmacological, cognitive behavioral, and behavioral therapy. Even while several studies have shown that music and social connection can improve dementia symptoms, just nine studies have coupled music with other activities to accomplish. These other activities included playing games, performing crossword puzzles, gardening, and engaging in physical and mental activities. This study has demonstrated that integrating social interactions can be enhanced by MT. 
